# Exploring secondary prevention after stroke: a survey of Irish stroke clinical nurse specialists and advanced nurse practitioners

**DOI:** 10.1007/s11845-025-04266-y

**Published:** 2026-01-13

**Authors:** Sarah-Jane Byrne, David Williams, Declan Patton, Frances Horgan

**Affiliations:** 1 Department of Geriatric and Stroke Medicine, Beaumont Hospital, Dublin 9, Ireland; 2 Department of Geriatric and Stroke Medicine, Royal College of Surgeons in Ireland, University of Medicine and Health Sciences , Dublin, Ireland; 3 Wounds and Trauma Research Centre, School of Nursing and Midwifery, Royal College of Surgeons in Ireland , University of Medicine and Health Sciences, Dublin, Ireland; 4 School of Physiotherapy, The Royal College of Surgeons in Ireland, University of Medicine and Health Sciences, Dublin, Ireland

**Keywords:** Advanced nurse practice, Nursing education, Nursing methods, Secondary prevention, Stroke

## Abstract

**Background:**

ESD enables stroke patients to leave hospital earlier and continue receiving nursing and therapy at home over a 6–8-week period. The CNS role within ESD is relatively new, and its involvement in stroke secondary prevention is not well defined.

**Aims & objectives:**

To describe the current role, knowledge, and practice of Stroke Clinical Nurse Specialists (CNSs) and Advanced Nurse Practitioners (ANPs) in secondary prevention and their contribution to early supported discharge (ESD) for stroke patients.

**Design:**

Cross-sectional survey.

**Methods:**

An online survey was distributed to Stroke CNSs and ANPs working in inpatient stroke services or ESD teams. It explored three domains: (i) secondary prevention at initial hospital contact, (ii) secondary prevention at discharge, (iii) secondary prevention during ESD.

**Results:**

The respondents described the pivotal role of stroke clinical nurse specialists (CNSs) and advanced nurse practitioners (ANPs) in delivering secondary prevention from hospital admission through to discharge. Persistent challenges in patient information retention during acute care were highlighted. Participants identified structured national resources—particularly printed materials over apps—as essential tools for effective secondary prevention.

**Conclusion:**

Despite broad recognition of their value, integration of stroke CNSs into Early Supported Discharge (ESD) teams remains inconsistent, signalling a key area for service development in Ireland. There is a clear need to enhance continuity of nurse-led secondary prevention in community settings, particularly within ESD pathways.

**Supplementary Information:**

The online version contains supplementary material available at 10.1007/s11845-025-04266-y.

## Introduction

Stroke is a leading cause of global mortality and morbidity, affecting over 15 million people annually [1, 2]. Effective secondary prevention requires identifying the clinical mechanism of the initial stroke and optimising modifiable risk factors such as hypertension, hyperlipidaemia, and lifestyle behaviours [1, 3, 4]. While definitions vary, some exclude pharmacological and surgical interventions [5]. Stroke nurse specialists within Early Supported Discharge (ESD) teams play a key role in managing these risks through both lifestyle education and medication support.

Despite evidence that up to 90% of strokes are preventable through lifestyle changes [6], many survivors struggle with poorly controlled risk factors and face significant challenges transitioning to community living [7, 8]^.^ Unmet needs post-discharge are common among patients and families [7–10]. ESD models, which reduce hospital stays and improve recovery [9–13, 14], offer multidisciplinary care at home over 6–8 weeks [15–19]. However, the specific role of the stroke clinical nurse specialist within ESD remains underexplored [20].

Nurses are central to secondary prevention, engaging patients in risk factor modification and supporting adherence [21]. Yet, workload pressures may hinder consistent delivery [5]. European surveys show variability in stroke nursing development across countries [22]. In Ireland, stroke CNSs and ANPs contribute to inpatient care and education, as outlined in national guidelines [23], but post-discharge support remains fragmented [6, 20, 24]. There is limited evidence on how community-based nurse-led secondary prevention can be structured within Irish ESD services [22].

This study aims to explore current practices of Irish stroke CNSs and ANPs in secondary prevention, to inform the development of a community-integrated ESD model.

## Methods

### Study design

We employed a cross-sectional survey design and used an online survey (platform SurveyMonkey). We followed the Consensus-based Checklist for Reporting of Survey Studies(CROSS)(www.equator-network.org/reporting-guidelines/a-consensus-based-checklist-for-reporting-of-survey-studies-cross/).

### Survey instrument

Data was collected using a purpose-designed questionnaire developed specifically for stroke Clinical Nurse Specialists (CNS) and Advanced Nurse Practitioners (ANP). The survey was adapted for the Irish context from previous UK and European surveys [20, 22, 25]. The survey comprised 31 items organised into four sections: (A) Demographic Data, (B) Secondary Prevention at First Patient Contact, (C) Secondary Prevention on Discharge from Hospital, and (D) Early Supported Discharge.

**Section A: Demographic Data** (Questions 1–3) collected information on respondents’ current nursing position, gender, and years of experience in stroke care.

**Section B: Secondary Prevention at First Patient Contact** (Questions 4–14) examined the timing and type of initial patient contact, discussion of stroke diagnosis and warning signs, assessment of pre-stroke lifestyle behaviours, and education regarding secondary prevention lifestyle factors.

**Section C: Secondary Prevention on Discharge from Hospital** (Questions 15–26) assessed current practices for providing secondary prevention education at discharge, including pre-discharge information sessions, satisfaction with current services, patient follow-up contact patterns, and referral practices to other healthcare professionals. Questions also explored preferences for educational resources.

**Section D: Early Supported Discharge** (Questions 27–31) evaluated the presence and composition of ESD teams, awareness of stroke CNS roles within ESD, and perceived value of CNS involvement in community-based secondary prevention.

The survey employed multiple response formats including multiple-choice questions, select-all-that-apply options, and open-ended questions requesting detailed explanations or suggestions. Prior to survey administration, participants were provided with an embedded patient information leaflet and consent form.

### Sample

The target sample were stroke clinical nurse specialists and advanced nurse practitioners working in Ireland. In Ireland, Clinical Nurse Specialists must hold an honours nursing degree, be registered with the NMBI, have at least two years of specialty experience, and possess a relevant postgraduate qualification at Level 8 or higher [26]. Stroke CNS and ANPs in this survey would have met these criteria to hold their specialist roles.

The gatekeeper for the study was the Nurse Lead at the Irish National Stroke Programme to contact the intended participants. Stroke clinical nurse specialists and advanced nurse practitioners in hospital and community settings were contacted by the Nurse Lead. There were approximately 40 stroke CNS’s and ANP’s on this list at the time of the study.

### Pilot study

The survey was piloted in July 2023 with two stroke clinical nurse specialists and advanced nurse practitioners to refine any questions and ascertain the time taken to complete. Some minor revisions were made to the survey.

### Data collection

#### Procedure

The gatekeeper disseminated the survey invitation in August 2023. The participant information leaflet and consent form will be embedded in the survey. Participants were given two months, to complete this survey and two email reminders were sent.

Individual survey invitations were sent via SurveyMonkey’s email collector to eligible stroke CNS participants. Each email address was linked to a unique survey URL that could only be accessed once, preventing duplicate responses. The system automatically tracked completed surveys and prevented resubmission.

Unique visitors were identified through SurveyMonkey’s individual email invitation system, which assigned a unique survey link to each participant. Of the 50 stroke CNS professionals invited, 30 (60%) accessed the survey (view rate), 23 (46%) provided consent and initiated the survey (participation rate), and 20 (40%) completed all survey sections (completion rate), yielding an overall response rate of 40%.

### Ethical considerations

The study was approved by RCSI ethics committee REC202303018. All participants provided informed consent.

### Data preparation and analysis

Descriptive statistics were used to summarise data. Frequencies and percentages were used to describe the demographic characteristics of the participants regarding their role, gender and years of experience and key survey variables regarding the participants nursing practice in three areas.

## Results

### Demographic data

**Fig. 1:**
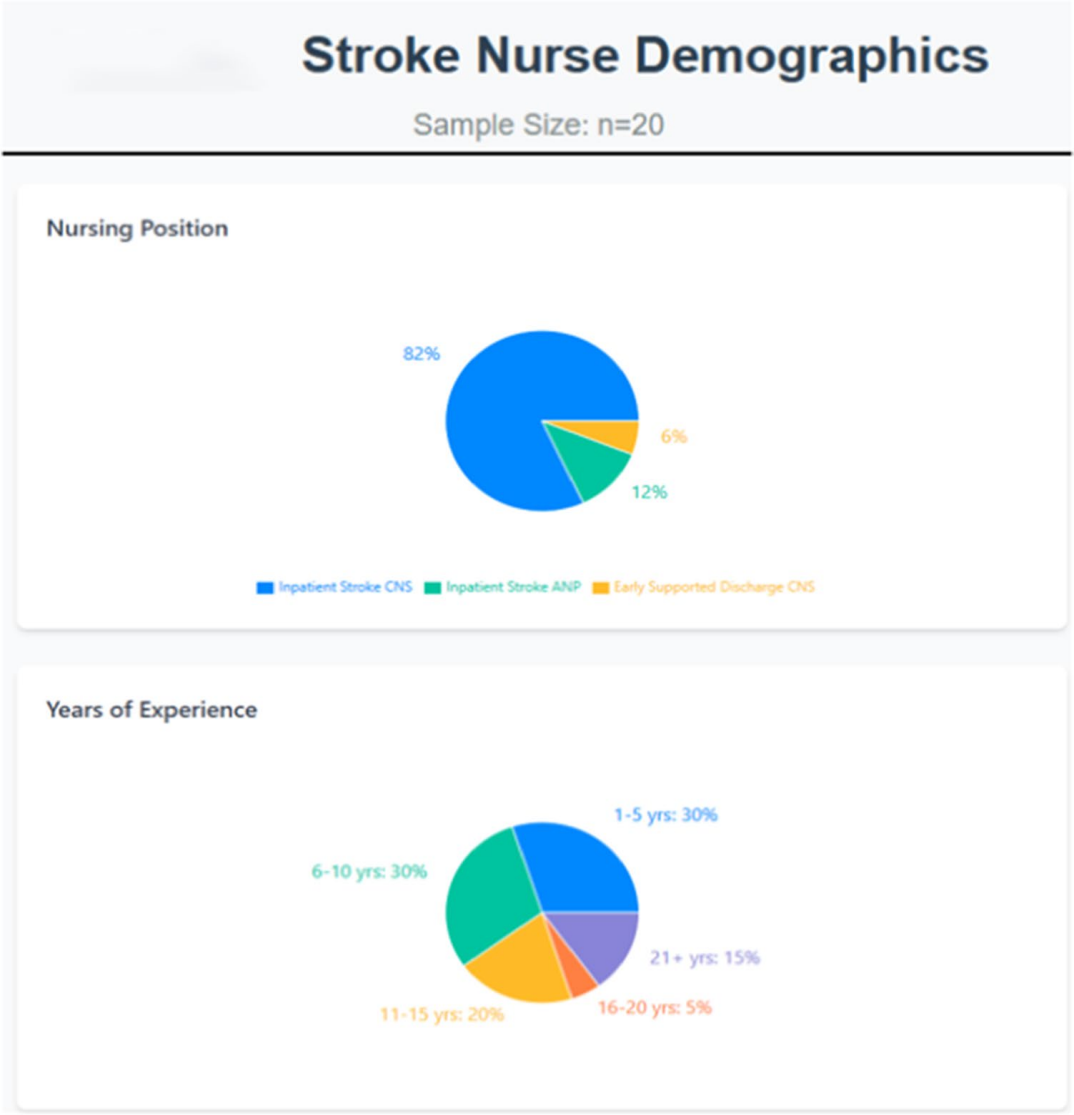
Stroke nurse demographics

There was a 50% response rate (*n* = 20). 82% (*n* = 16) were inpatient stroke clinical nurse specialists, 6% were early supported discharge stroke clinical nurse specialists and 12% (*n* = 3) were inpatient stroke advanced nurse practitioners. All respondents were female with a varied clinical experience as shown in Fig. 1:

### Secondary prevention at first patient contact in hospital

Table 1 outlines where nurse specialists first meet patients. Most initial contact occurs in the Emergency Department (70%, *n* = 14), followed by within 24 h of admission (25%, *n* = 5), and rarely in community settings (5%, *n* = 1). Overall, 95% (*n* = 19) report first contact in hospital, with only 5% (*n* = 1) via home visit.

**Table 1:** Survey results for stroke CNS/ANP education and lifestyle discussions (*n* = 20)

Question and response options	% (*n* =)	% (*n* =)
Secondary Prevention at First Patient Contact in Hospital		
When do you have contact with the patient for the first time?		
On arrival to ED	70% (*n* = 14)	
Within 24 h	25% (*n* = 5)	
Other: Community	5% (*n* = 1)	
What type of contact do you have with the patient for the first time?		
Inpatient	95% (*n* = 19)	
Home Visit	5% (*n* = 1)	
Do you discuss the patient’s stroke diagnosis with them?		
Yes	85% (*n* = 17)	
No	0%	
Sometimes	15% (*n* = 3)	
Do you discuss signs of stroke with the patient?		
Yes	90% (*n* = 18)	
No	5% (*n* = 1)	
Sometimes	5% (*n* = 1)	
Which signs of stroke do you discuss?		
Balance	75% (*n* = 15)	
Visual Disturbances	70% (*n* = 14)	
Facial Droop	95% (*n* = 19)	
Limb Weakness	95% (*n* = 19)	
Speech Disturbance	95% (*n* = 19)	
No, I do not discuss	5% (*n* = 1)	
What resources do you use when discussing signs of stroke?		
IHF Booklets	70% (*n* = 14)	
Locally formatted information leaflets	15% (*n* = 3)	
Croi, Google Images, YouTube Videos	5% (*n* = 1) each	
Do you discuss stroke secondary prevention lifestyle?	**Pre-Stroke Behaviours**	**Post-Stroke Behaviours**
Yes	90% (*n* = 18)	95% (*n* = 19)
No	5%(* n* = 1)	5% (*n* = 1)
Sometimes	5% (*n* = 1)	
What lifestyle behaviours do you discuss?	**Pre-Stroke Behaviours**	**Post-Stroke Behaviours**
Smoking	90% (*n* = 18)	100% (*n* = 20)
Alcohol	90% (*n* = 18)	100% (*n* = 20)
Stress	75% (*n* = 15)	65% (*n* = 13)
Nutrition	65% (*n* = 13)	80% (*n* = 16)
Unprescribed Drug Use	60% (*n* = 12)	55% (*n* = 11)
Physical Activity	80% (*n* = 16)	90% (*n* = 18)
No, I don’t discuss	10% (*n* = 2)	
Do you discuss medication compliance as part of secondary prevention?		
Yes	95% (*n* = 19)	
Sometimes	5% (*n* = 1)	
Do you offer a Secondary Prevention Session Pre Discharge		
Yes	85% (*n* = 17)	
Duration of the Session	10-15 min	
Secondary Prevention on Discharge from Hospital		
Discharge Resources Provided		
IHF Booklet	80% (*n* = 16)	
Stroke Passport	5% (*n* = 1)	
Stroke Information Folder	5% (*n* = 1)	
No Resource Provided	10% (*n* = 2)	
Onward Referral Services		
IHF Stroke Connect Service	85% (*n* = 17)	
Smoking Cessation Nurse	75% (*n* = 15)	
Public Health Nurse	50% (*n* = 15)	
ESD Nurse	45% (*n* = 14)	
Alcohol Liaison Nurse	45% (*n* = 14)	
Booklet or App?		
Booklet	95% (*n* = 19)	
App	5% (*n* = 1)	
Do patients contact you in the first 6 months following their discharge for secondary stroke prevention information?		
Yes	20 (*n* = 4)	
No	30 (*n* = 6)	
Sometimes	50% (*n* = 10)	
Secondary Prevention in Early Supported Discharge		
Do you have an ESD Team in your hospital?		
Yes	70% (*n* = 17)	
No	30% (*n* = 6)	
Is there a Stroke CNS as part of your ESD Team?		
Yes	40% (*n* = 8)	
No	60% (*n* = 14)	
Are you aware of the role the Stroke CNS has as part of the ESD Team?		
Yes	80% (*n* = 18)	
No	20% (*n* = 4)	
Do you think a phone-call/house visit from a Stroke CNS would be useful?		
All Stroke Patients	70% (*n* = 16)	
ESD Patients Only	30% (*n* = 6)	
What value does the Stroke CNS in ESD provide in secondary prevention in the community?		
Education on Medication Compliance	100% (*n* = 20)	
Education on Lifestyle Behaviours	100% (*n* = 20)	
Signposting patients to other community services	100% (*n* = 20)	
Follow-up link to inpatient Stroke CNS	90% (*n* = 18)	
How long should CNS home visits be?		
30 min	45% (*n* = 9)	
45 min	45% (*n* = 9)	
60 min	10% (*n* = 2)	

Stroke diagnosis is discussed early, with 85% (*n* = 17) consistently engaging patients. Warning signs are also prioritised, 90% (*n* = 18) regularly address them. The most cited signs are facial droop, limb weakness, and speech disturbance (each 95%, *n* = 19), followed by balance issues (75%, *n* = 15) and visual disturbances (70%, *n* = 14). To support these discussions, 70% (*n* = 14) use Irish Heart Foundation (IHF) booklets, 15% (*n* = 3) use local leaflets, and 5% (*n* = 1) use Croí and multimedia resources such as, Google Images, or YouTube.

Qualitative responses highlight additional early topics: stroke definition and investigations, blood pressure control, heart rhythm, treatment plans, rehabilitation, and work-life balance. Referrals to alcohol liaison nurses may also occur. Nurse specialists answer follow-up questions after consultant discussions. Medication management is addressed by 95% (*n* = 19) during hospital stay, with further qualitative insights detailed in Table 2. One nurse specialist noted avoiding detailed discussions during inpatient care to prevent information overload. Instead, they send materials post-discharge or follow up by phone.

**Table 2:** Qualitative responses of nurse specialists

Variable	Response
Do you discuss medication compliance as a secondary prevention topic with the patient?	• Review of previously prescribed medications • Routine for taking these medications • Factors contributing to their adherence or non-adherence • Patient understanding of medications and potential common side effects • How the patient renews their prescription also
Would a specific Stroke Secondary Prevention Booklet or online App be helpful for you to provide to patients on discharge?	• App for younger stroke patients • Dependent level of engagement with smart devices • Availability of a smart device • Language barriers if booklets in only one language
Barriers to Stroke Secondary Prevention Provision	• Large inpatient admission numbers • Time constraints in the inpatient setting • Lack of private spaces to talk to patients regarding sensitive topics • Concerns that the information provided is not sufficient • Service delivery is informal and not structured on a national or local level • Patients are not ready to receive information as inpatient
Do patients contact you in the first 6 months following their discharge for stroke secondary prevention information?	• Patient contact includes new issues, including where to get a new prescription, follow up appointments with consultant. questions about symptoms, blood pressure and medications • Patients’ family members looking for information and advice
What are the factors that influence you to refer to another nursing professional?	• If they require a specific service that is not related to stroke, The availability of a service and their own time constraints if they cannot provide the information to the patient directly • Reduced time and resources to provide information as an inpatient • Patients’ interest, their ability to engage with services post stroke have an impact as some patients’ disability may be a factor which will reduce their ability to engage
Is there any other information that you would like to see in the Stroke Booklet or information that was included/not included in a Stroke Booklet that you have already?	• Risk of re-occurrence of stroke • Information on continence and pain • Information on social prescribing and community activities • Information on returning to work • A simple explanation of hospital investigations • Information on all medications that may be prescribed, the risks associated with non-adherence and making informed choices • The importance of dialling 112 or attending the emergency department immediately if signs of stroke present, not to wait or attend the general practitioner first
What value do you think the Stroke CNS in ESD has in providing Stroke Secondary prevention to patients in the community?	• Advocate for individual issues that any patient has • Secondary prevention • Health promotion within the household • Education and support of all affected by the stroke diagnosis • Individuals may reveal more about their lifestyle in their own home environment that they would in hospital • Education on medication compliance, lifestyle behaviours • Signposting to other community services • Follow-up link to the inpatient clinical nurse specialists

### Secondary prevention on discharge from hospital

**Fig. 2:**
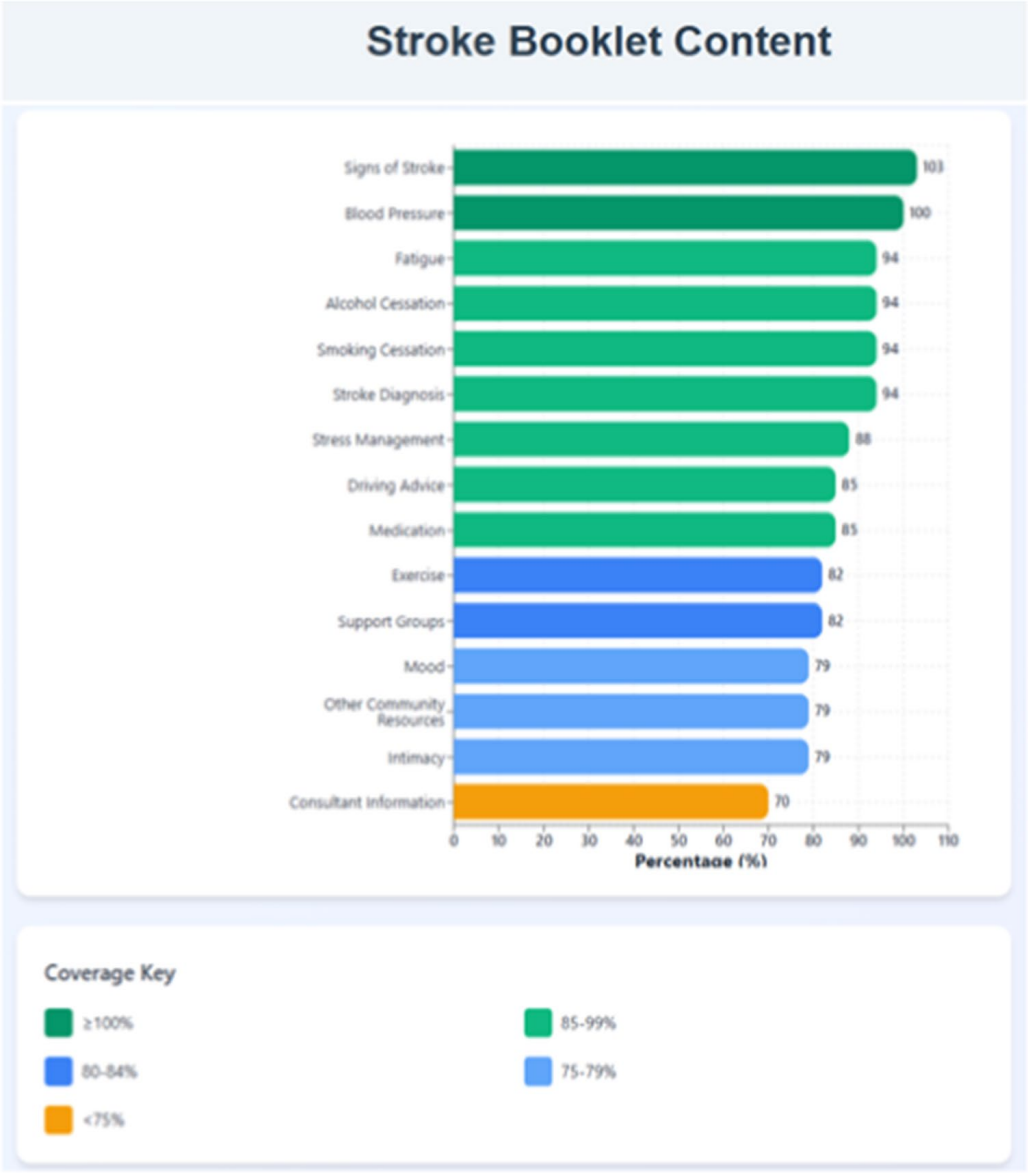
Stroke booklet content

Respondents were asked to rate current delivery of stroke secondary prevention information to patients on their transition from hospital to home. On a scale of zero (not satisfied) to ten (satisfied), they reported an average level of 5.5. Areas of improvement are highlighted in Table 2 with lack of a local and national structure and an aid to help them deliver secondary prevention such as an educational booklet or an app. Reasons for choosing a booklet over an app to aid secondary prevention information delivery can be found in Table 2. Due to the age profile and demographics of the patients, most respondents report that a booklet would be beneficial to them to provide to patients on discharge (Table 1). The content nurse specialists would like to see in an educational booklet are shown on Fig. 2 with additional content ideas on Table 2. On discharge, factors that influence the stroke nurse specialists to refer to other nursing professions include, patient need, and, in their experience, they find that patients are more receptive to secondary prevention information after the initial acute phase therefore more likely to hold information better outside the acute hospital environment as highlighted in Table 2.

### Secondary prevention in early supported discharge

Although 70% (*n* = 17) of respondents’ report having an early supported discharge team in their hospital only 40% (*n* = 8) have an ESD CNS on their ESD team (Table 1). 80% (*n* = 18) of the stroke nurse specialists report being aware of the role of the stroke nurse specialist as part of an early supported discharge team. They responded that all stroke patients should receive a phone-call or home visit from the stroke CNS on the ESD team with home visits being an average of 45 min as shown in Table 1. The respondents provided narrative details on what they viewed as the value of the clinical nurse specialist role as part of an early supported discharged team (Fig. 2).

## Discussion

This study described the current practice of stroke nurse specialists both CNSs and ANPs in initiating secondary prevention from early hospital contact through to discharge. While education typically begins within 24 h in acute settings, patients often struggle to retain information due to cognitive overload and environmental stressors [27, 28]. To mitigate this, nurses adopt follow-up strategies such as phone calls and postal delivery of printed materials, reflecting a need for adaptable, patient-centred communication. These findings align with the UK and Irish National Clinical Guidelines for Stroke (2023), which advocate for early, structured education and continuity of care across the stroke pathway [23]. However, unlike stroke services in Sweden and the Netherlands, where nurse-led follow-up is systematically embedded within national stroke care pathways [29, 30], the Irish context reveals a more ad hoc approach, with individual nurses developing compensatory strategies in the absence of standardised protocols [31].

Despite efforts to educate patients during hospitalisation, discharge practices remain inconsistent, and post-stroke care is often fragmented due to the absence of a coordinated national framework [32, 33]. This fragmentation is particularly concerning given that Ireland lacks the integrated stroke care networks seen in countries such as Germany and Denmark, where regional stroke centres are linked through coordinated referral and follow-up systems [34–36]. Nurse specialists identified the need for standardised, age-appropriate resources, with printed booklets preferred over digital formats. This preference contrasts with the increasing digital literacy and telehealth adoption observed in Scandinavian countries, suggesting that the Irish stroke population—or healthcare infrastructure—may require tailored, non-digital solutions to ensure equitable access [34–36]. These materials should include guidance on medication, lifestyle changes, diagnosis, and rehabilitation, and be available in multiple languages to support diverse patient needs. Integration of CNSs into ESD teams is inconsistent, although respondents emphasised its value in supporting hospital-to-home transitions. Home visits and follow-up calls—typically lasting 30 to 60 min—were found to improve adherence and risk factor control [37, 38]. Addressing modifiable lifestyle-related risk factors remains central to secondary prevention [39], and CNSs are well-positioned to lead this work in the community.

Overall, this study identifies practical and systemic challenges in Irish stroke care and reveals opportunities for service development. Establishing structured national guidelines, ensuring consistent integration of CNSs into ESD teams, and providing standardised educational resources could enhance the quality and impact of stroke secondary prevention. These findings can help to inform national policy and redesigning community stroke services to better support patient outcomes and facilitate earlier, safer reintegration into the community [40]. Further research into patient experiences would help refine the CNS role in secondary prevention.

## Limitations

The small sample size limits the generalisability of the findings. As participation was voluntary, there is also a risk of self-selection bias, whereby individuals with stronger opinions or more positive experiences may have been more likely to respond. An additional limitation is the reliance on self-reported data, which may have introduced recall bias and affected the accuracy of the reported information, resulting in an overrepresentation of certain perspectives and underrepresentation of others. As an exploratory study, the questionnaire served to elicit broad perspectives rather than measure latent constructs requiring validity testing. Content validity was established through pilot testing, which was deemed sufficient for the study’s aims. The questionnaire comprised factual and categorical items not intended to form scales, thus internal consistency testing was not applicable. These factors should be considered when interpreting the results and drawing conclusions.

## Strengths

This study demonstrates a number of key strengths. Ethical integrity was upheld including approval from the relevant Research Ethics Committee (REC). To our knowledge this is the first survey conducted among Irish clinical nurse specialists and advanced nurse practitioners on their practice of stroke secondary prevention. The use of a mixed-methods design, combining quantitative and qualitative data, enabled a rich understanding of stroke clinical nurse specialists experiences and preferences regarding stroke secondary prevention. The findings in this survey have the potential to inform an educational tool for use by stroke nurse specialists in ESD in the community with stroke patients.

## Conclusions

The survey findings highlight the important role of stroke nurse specialists in delivering secondary prevention from the point of first hospital contact through to discharge. Challenges persist in patient information retention, prompting alternative approaches post-discharge to support delivery. The findings highlight the role of stroke CNS and ANPs in ESD in the community as a key resource to optimise secondary prevention outcomes.

## Supplementary Information

Below is the link to the electronic supplementary material.

Supplementary file1 (DOCX 21 KB)

Supplementary file2 (PDF 150 KB)

## Data Availability

The data supporting the conclusions of this article are included within the article. Aggregated data are available upon request.
