# Supplementary material for: Exploring secondary prevention after stroke: a survey of Irish stroke clinical nurse specialists and advanced nurse practitioners
Source: Ir J Med Sci. 2026 Jan 13;195(2):1011–20. doi: 10.1007/s11845-025-04266-y (PMC13190491; doi:10.1007/s11845-025-04266-y)
Supplement: Supplementary file 2 — Supplementary file2 (PDF 150 KB) [file 11845_2025_4266_MOESM2_ESM.pdf]

Survey For Stroke Clinical Nurse Specialists

Clinical Nurse Specialist practice in a speciality area of nursing and encompasses a major clinical focus of care to patients or clients and their families in hospital, community and outpatient settings. (HSE, 2021).

The Patient Information Leaflet and Consent will be embedded here at the start of the survey.

Section A: Demographic Data

1. What is your current nursing position?
   a. Inpatient Stroke CNS
   b. Early Supported Discharge Stroke CNS
   c. Inpatient Stroke ANP
   d. Other: please specify
2. What is your gender?
   a. Male
   b. Female
   c. Other
   d. Prefer not to specify
3. How many years of experience do you have working in stroke care?
   a. 1-5 years
   b. 6-10years
   c. 11-15years
   d. 16-20years
   e. 21+ years

Section B: Secondary Prevention at first Patient Contact

4. When do you have contact with the stroke patient for the first time?
   a. On arrival to the Emergency Department
   b. Within 24hours of Stroke
   c. Other, please specify:
5. What type of contact do you have with the stroke patient for the first time? Select all that apply
   a. Inpatient
   b. Telephone
   c. Home Visit
   d. Other:
6. As part of your role do you discuss the patients Stroke diagnosis with them?
   a. Yes
   b. No
   c. If yes, please provide details on what you discuss and what resources you use:
7. As part of your role do you discuss signs of stroke with the patient?
   a. Yes
   b. No
8. Which of the following signs of stroke do you discuss. Select all that apply.
   a. Balance
   b. Visual Disturbances
   c. Facial Droop
   d. Limb Weakness

    e. Speech Disturbances
    f. No, I do not discuss the signs of stroke.
9. What resources do you use when discussing signs of stroke with patients?
    a. Answer:
10. Do you ask the patients about their pre-stroke lifestyle behaviours as part of your role?
    a. Yes
    b. No
11. If yes to Q10, please select all pre-stroke lifestyle behaviours that apply when you discuss with the patient as part of your role.
    a. Smoking
    b. Alcohol
    c. Stress
    d. Nutrition
    e. Unprescribed Drug Use
    f. No, I do not discuss with the patient.
12. Do you discuss stroke secondary prevention lifestyle factors/behaviours with the patient as part of your role?
    a. Yes
    b. No
13. If yes to Q12, please select all stroke secondary lifestyle factors/behaviours that apply when you discuss with the patient as part of your role.
    a. Smoking
    b. Alcohol
    c. Stress
    d. Nutrition
    e. Unprescribed Drug Use
    f. Physical Activity
    g. No, I do not discuss with the patient.
14. Do you discuss medication compliance as a secondary prevention topic with the patient?
    a. Yes, please explain what is discussed:
    b. No

<u>Section C: Secondary Prevention on Discharge from Hospital</u>

15. Do you offer a secondary prevention information session pre-discharge to patients?
    a. Yes, please explain (what team members are involved, duration, what information is provided)
    b. No
16. Are you satisfied with the current service provided to patients on Stroke Secondary Prevention?
    a. Yes
    b. No, please explain how it could be improved:
17. Do patients contact you in the first 6 months following their discharge for stroke secondary prevention information?
    a. Yes, and reasons for contact:
    b. No
18. On a scale of 0-10, what is your level of satisfaction with the current delivery of stroke secondary prevention information to patient?
    a. Very Satisfied
    b. Satisfied
    c. Neutral
    d. Dissatisfied

e. Very Dissatisfied

19. How do you think the delivery of Stroke secondary prevention information could be improved following patient discharge?
    a. Answer:
20. In your current role do you refer patients to another nursing professional for stroke secondary prevention?
    a. Public Health Nurse
    b. ESD Stroke CNS
    c. Alcohol Liaison Nurse
    d. Smoking Cessation Nurse
    e. Irish Heart Foundation Stroke Connect Service
    f. Other:
21. What are the factors that influence you to refer to another nursing professional?
    a. Answer:
22. Do your patients receive a stroke booklet on discharge home?
    a. Irish Heart Foundation Booklet
    b. Hospital Discharge Booklet
    c. Stroke Secondary Prevention Booklet
    d. No, they do not receive anything.
23. Would a specific Stroke Secondary Prevention Booklet or online App be helpful for you to provide to patients on discharge?
    a. Booklet
    b. App
    c. Neither
24. Do you think patients would prefer a Stroke Secondary Prevention Booklet or to download an App and why?
    a. Answer:
25. What information from the list below would you like to see in the stroke booklet? Please select all that apply:

Stroke Diagnosis	Signs of Stroke	Smoking Cessation	Alcohol Cessation	Information on Blood Pressure
Information on Stress	Information on Diet	Information on Mood	Information on Fatigue	Information on Exercise
Information on intimacy post-stroke	Information on Exercise post-stroke	Medication Information	Follow Up Appointment with Consultant	Support Group Information

26. Is there any other information that you would like to see in the Stroke Booklet or information that was included/not included in a Stroke Booklet that you have already?
    a. Answer:

<u>Section D: Early Supported Discharge</u>

27. Do you have an ESD Team in your hospital?
    a. Yes
    b. No
28. Is there a Stroke CNS as part of your ESD Team?
    a. Yes
    b. No
29. Are you aware of the role the Stroke CNS has as part of the ESD Team?
    a. Yes

    b. No

30. Do you think a phone-call/house visit from a Stroke CNS for all stroke patients or just patients on Early Supported Discharge would be useful?
    a. All Stroke Patients
    b. ESD Patients only
31. What value do you think the Stroke CNS in ESD has in providing Stroke Secondary prevention to patients in the community? Select all that apply.
    a. Education on Medication Compliance
    b. Education on Lifestyle Behaviours
    c. Signposting patients to other community services
    d. A follow up link to Inpatient Stroke CNS'
    e. Other, please specify:

Thank you and end of survey
